# Characteristics of HIV seroprevalence of visitors to public health centers under the national HIV surveillance system in Korea: cross sectional study

**DOI:** 10.1186/1471-2458-9-123

**Published:** 2009-05-05

**Authors:** Mee-Kyung Kee, Jin-Hee Lee, Chaeshin Chu, Eun-Ju Lee, Sung-Soon Kim

**Affiliations:** 1 Division of AIDS, Center for Immunology and Pathology, Korea National Institute of Health, Korea Centers for Disease Control and Prevention, Seoul, Republic of Korea; 2 Division of Epidemic Intelligence Service, Korea Centers for Disease Control and Prevention, Seoul, Republic of Korea

## Abstract

**Background:**

In Korea, the cumulative number of HIV-infected individuals was smaller than those of other countries. Mandatory HIV tests, dominating method until 1990's, have been gradually changed to voluntary HIV tests. We investigated HIV seroprevalence status and its characteristics of visitors to Public Health Centers (PHCs), which conducted both mandatory test and voluntary test under the national HIV/STI surveillance program.

**Methods:**

We used HIV-testing data from 246 PHCs in 2005 through the Health Care Information System. The number of test taker was calculated using the code distinguished by the residential identification number. The subjects were classified into four groups by reason for testing; General group, HIV infection suspected group (HIV ISG), HIV test recommended group (HIV TRG), and sexually transmitted infection (STI) risk group.

**Results:**

People living with HIV/AIDS were 149 (124 male and 25 female) among 280,456 individuals tested at PHCs. HIV seroprevalence was 5.3 per 10,000 individuals. Overall, the male revealed significantly higher seroprevalence than the female (adjusted Odds Ratio (adj. OR): 6.2; CI 3.8–10.2). Individuals aged 30–39 years (adj. OR: 2.6; CI 1.7–4.0), and 40–49 years (adj. OR: 3.8; CI 2.4–6.0) had higher seroprevalence than 20–29 years. Seroprevalence of HIV ISG (voluntary test takers and cases referred by doctors) was significantly higher than those of others. Foreigners showed higher seroprevalence than native Koreans (adj. OR: 3.8; CI 2.2–6.4). HIV ISG (adj. OR: 4.9; CI 3.2–7.5), and HIV TRG (adj. OR: 2.6; CI 1.3–5.4) had higher seroprevalence than General group.

**Conclusion:**

A question on the efficiency of current mandatory test is raised because the seroprevalence of mandatory test takers was low. However, HIV ISG included voluntary test takers was high in our result. Therefore, we suggest that Korea needs to develop a method encouraging more people to take voluntary tests at PHCs, also to expand the anonymous testing centers and Voluntary Counselling and Testing Program (VCT) for general population to easily access to HIV testing.

## Background

The first case of acquired immunodeficiency syndrome (AIDS) in Korea was a foreign resident, and the first human immunodeficiency virus (HIV)-infected Korean contracted the virus during overseas travel in 1985 [1]. This stimulated a national preventative system for this emerging infection in Korea, which became prevalent in the United States and Europe in the early 1980s [2,3]. In 1985, mandatory HIV tests were established in commercial sex workers (CSWs) with sexual contact with foreigners [1,4], and in 1986 mandatory test was expanded to CSWs with sexual contact with Koreans and hemophiliacs. Prison inmates and donated blood samples were included in the mandatory testing group in July 1987 [5]. HIV mandatory testing was extended to seafarers with long-term overseas contracts in 1988 [6], and voluntary anonymous HIV testing was allowed to ensure broader screening in 1989. Article 8 of the Prevention Act for Acquired Immunological Deficiency Syndrome (1986) established the national surveillance system for the detection of HIV-positive individuals. Public health centers (PHCs) were instructed to conduct HIV screening tests for sexually transmitted infection (STI) risk groups periodically, and for volunteer groups arbitrarily [7]. The number of mandatory HIV tests in Korea reached approximately 2 million until 1997, with the addition of hair salon employees, restaurant employees, and food industry employees, food industry sanitation workers and public health personnel. However, in 1998 the Korean government amended the HIV testing policy from mandatory testing to voluntary testing by the Act of Health Check for Food Industry Employees and Others. This exempted restaurant, food industry employees and others from mandatory HIV testing [8].

UNAIDS estimated 40.3 million people living worldwide with HIV/AIDS in 2005, and AIDS-associated deaths were up to 20 million [9]. HIV/AIDS has been expanding rapidly in India, China, and Southeast Asia. China has increased recently with an estimated 650,000 infected individuals [10]. The seroprevalence in Russia increased more than five times from 1997 to 2002 [11]. Frequent visits between Korea and these countries might have increased the risk of individuals spreading HIV/AIDS in Korea [12]. The percentage of HIV infections in Korean adults aged 19–45 years as estimated by the World Health Organization (WHO) in 2003 was relatively low at 0.01% [13]. A total of 4,341 HIV infections were identified in Korea from 1985 to 2005, including 88% Korean and 12% foreigners. The proportion of HIV infections in the male (89%) was much higher than that in the female (11%) [14]. The number of newly diagnosed HIV infections has increased yearly in Korea since 1998: 137 in 1998, 244 in 2000, 457 in 2002, and 763 in 2004 [14]. The Korean government conducted various projects to slow down the increasing HIV infection; HIV diagnosis and financial support of treatment were offered for people living with HIV/AIDS (PLWHA) [14]. There are no epidemiological studies to estimate or project the rate of HIV transmission and to evaluate HIV/AIDS prevention projects in Korea, even any investigation about essential HIV seroprevalence. As the one of HIV testing centers in Korea, PHCs are responsible to implement HIV/STI prevention for the susceptible, low-income individual, and the anonymous, through taking both HIV mandatory testing and voluntary testing under national HIV surveillance system. In 2005, PHCs conducted about 6% of HIV tests in Korea, and detected approximately 19% of newly diagnosed HIV infections [15]. It was urgent to identify the status of HIV seroprevalence among visitors to PHCs whose HIV positivity was higher than other HIV test centers. Therefore, we investigated HIV seroprevalence status and its characteristics of visitors to PHCs.

## Methods

### Data Collection

PHCs in Korea have adopted a nationally incorporated computerized data processing system, known as the health care information system (HCIS), since 2000. The system handles various health-related test results along with other demographic and laboratory information. A total of 372,692 HIV tests were carried out at 246 PHCs in 2005. The following data from the HCIS were collected from each center: institutional identification, reception number, reception date, gender, date of birth, residence areas, test method, test kit, test result, reason for testing, and code. The code is a parameter to identify the testing frequency of each individual, and it is linked to each residential identification number (RID, 13 digit numbers), unique number for each Korean, which is coded by the date of birth, gender, birth place and check number. The RID were declared during initial testing and in subsequent tests the unique code was internally assigned inside HCIS. The RID cannot be deduced from the code and it ensures an individual's confidentiality.

### Public HIV testing system under the National HIV surveillance

In Korea, HIV reactive samples from primary screening tests at PHCs are referred to local Institute of Health & Environment (IHE) for confirmatory testing. There are 17 local IHEs in 7 cities and in 10 provinces. Confirmed positive samples are referred to the division of AIDS at the Korean Centers for Disease Control and Prevention (KCDC), and the division makes final decision on HIV infection status for each sample [7].

### Subjects

All individuals in this study took free of charge HIV testing for personal physical examinations in community health improvement programs by PHCs. There were 14 reasons for HIV testing by the HCIS, and these were classified into four groups: The General group, the HIV infection suspected group (HIV ISG), the HIV test recommended group (HIV TRG), and the STI risk group. We grouped the STI risk group and the HIV TRG according to the "Guidelines for HIV/AIDS control" [7]. Individuals in the General group took HIV test as a part of health checkup, and the HIV ISG took HIV test based on suspicion of HIV infection (Table 1). As anonymous tests could not determine the number of individuals, it was not included in the seroprevalence estimates. We calculated the positivity for the anonymous testing group.

**Table 1:** Classification of the reason for HIV testing of visitors to public health centers in Korea (2005).

Class	Reason for HIV testing	Regulation
General group	**- Health checkup: **a comprehensive physical examination **- Medical certificate: **to apply for workplace permits, residence halls, licenses, officers, workers, welfare centers, detention centers and in shelters for women. **- Prenatal checkup** **- Others: **Employees of motels, restaurants, and beauty salons, etc.	Voluntary testing (but medical certificate for a job or residence appeared to be by routine testing.).

HIV infection s uspected group (HIV ISG)	**- Referral by doctor: **to take additional HIV tests when doctors suspect HIV infection at diagnosis **- Voluntary test taker: **to take HIV test as identified if suspicious.	Voluntary testing (because doctors or themselves are suspicious about HIV infection).

HIV test recommended group (HIV TRG)	**- Tuberculosis patient** **- Prisoner** **- Partner of HIV-infected individual**	Though routine test regulated by "Guideline for HIV/AIDS control.

Sexually transmitted infection risk group (STI risk group)	**- Commercial sex worker(CSW)** **- Bar employee** **- Tea-room employee** **- Massage parlor** **- Others: **one of the above four categories, but not specified	Mandatory testing except male, regular mandatory HIV tests are taken every six months by "Guideline for HIV/AIDS control" to prevent the spread of HIV to general population.

### Data Analysis

HIV seroprevalence was defined as the number of confirmed PLWHA divided by total number of HIV-tested individuals during the study period. Initially indeterminate samples that were positive on follow-up were included as HIV positive samples, and the first test date was defined as the date of diagnosis.

Tests without full RID or with incorrect RID (19,252 tests), and anonymous cases (9,877 tests) were excluded from the initial 372,692 tests. The testing frequency of each individual in a single year was referred to as the repeated number. The mean of the repeated number was calculated in each group and by gender. HIV seroprevalence was expressed as the number of PLWHA per 10,000 individuals. Logistic regression analysis was performed to examine factors that were independently associated with HIV seroprevalence. The multi-variable models were fit adjusting for variables (gender, age group, nationality, region, and reason for testing). Statistical significance (p < 0.05) was defined at the 95% confidence interval. All statistical analyses were performed using SAS 9.1.

## Results

**Figure 1:**
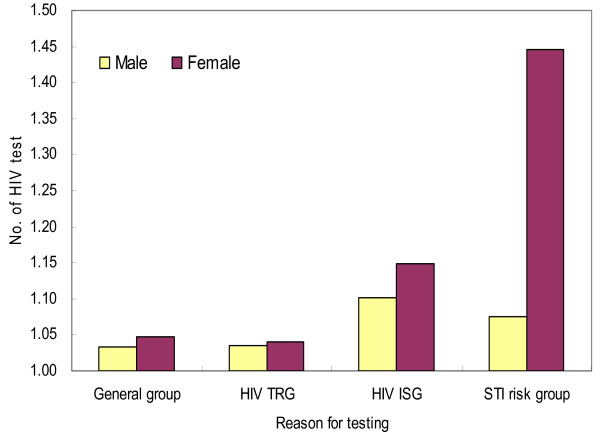
**The number of HIV test by gender and the group of reason for HIV testing (2005).** HIV TRG: HIV test recommend group. HIV ISG: HIV infection suspected group.  STI: sexually transmitted infection. Gender; male p<.0001, female p<.0001 Reason for HIV testing; General group P=0.1905, HIV TRG P=0.8971, HIV ISG P=0.0046, STI risk group P<0.0001

A total of 280,456 individuals were tested at 246 PHCs in 2005, and 149 PLWHA were identified. The repeated number was significantly greater in the STI risk groups than in other groups (p < 0.0001), and there were statistically significant differences in the repeated number between the male and the female in the HIV ISG and the STI risk groups, but not for the General group and the HIV TRG (Figure 1).

Table 2 presents demographic data about HIV seroprevalence in Korea. The female accounted for 68.7% of all tested individuals (n = 280,456), and twenties were the leading group (45.4%) while metropolitan city dweller took 40.4%. The STI risk group was outstanding (51.6%) in reason for testing. Overall HIV seroprevalence was 5.3 per 10,000 individuals and the male showed significantly higher seroprevalence than the female (adjusted Odds Ratio (adj, OR), 6.2; 95% Confidence Interval (CI), 3.8–10.2). HIV seroprevalence in the aged 30–39 years (adj. OR, 2.6; 95% CI, 1.7–4.0), and the aged 40–49 years (adj. OR, 3.8; 95% CI, 2.4–6.0) was higher than that in the aged 20–29 years as a reference. HIV seroprevalence in 9,959 foreigners was 18.1 cases per 10,000 individuals, and this was significantly higher than the seroprevalence among native Koreans (adj. OR, 3.8, 95% CI, 2.2–6.4). Small towns or rural areas revealed lower HIV seroprevalence than the metropolitan cities (adj. OR, 0.5; 95% CI, 0.4–0.7). The HIV ISG (adj. OR, 4.9; 95% CI, 3.2–7.5) and the HIV TRG (adj. OR, 2.6; 95% CI, 1.3–5.4) had higher seroprevalence than the General group. The lowest seroprevalence was seen in the STI risk group (1.5 per 10,000 individuals), but this was not significantly different from the General group (adj. OR, 1.1; 95% CI, 0.6–2.0) after adjusted by gender, age, nationality, and region.

**Table 2:** HIV seroprevalence of visitors to public health centers in Korea (2005).

	No. of HIV tested	No. of HIV tested individuals (%)	No. positive (%)	HIV Seroprevalence (95% CI)	Unadjusted OR (95% CI)	Adjusted OR (95% CI)
**Total**	343563	280456	149	5.3 (4.5,6.5)		
**Gender**						
Female	251483	192687 (68.7)	25 (16.8)	1.3 (0.8,1.8)	1.0	1.0
Male	92080	87769 (31.3)	124 (83.2)	14.1 (11.6,16.6)	10.9 (7.1,16.8)	6.2 (3.8,10.2)
**Age group**						
<20	17922	15587 (5.6)	4 (2.7)	2.6 (0.1,5.1)	0.9 (0.3,2.6)	0.6 (0.2,1.6)
20–29	165803	127393 (45.4)	35 (23.5)	2.7 (1.8,3.7)	1.0	1.0
30–39	86052	70723 (25.2)	56 (37.5)	7.9 (5.8,10.0)	2.9 (1.9,4.4)	2.6 (1.7,4.0)
40–49	43220	37559 (13.5)	41 (27.5)	10.9 (7.6,14.3)	4.0 (2.5,6.2)	3.8 (2.4,6.0)
50–59	16039	15196 (5.4)	8 (5.4)	5.3 (1.6,8.9)	1.9 (0.9,4.1)	1.4 (0.6,3.0)
60≤	14527	13998 (4.9)	5 (3.4)	3.6 (0.4,6.7)	1.3 (0.5,3.3)	0.9 (0.4,2.3)
**Nationality**						
Korean	333367	270497 (96.5)	131 (87.9)	4.8 (4.0,5.7)	1.0	1.0
Foreigner	10196	9959 (3.5)	18 (12.1)	18.1 (9.7,26.4)	3.7 (2.3,6.1)	3.8 (2.2,6.4)
**Region**						
Metropolitan	134613	113290 (40.4)	87 (58.4)	7.7 (6.1,9.3)	1.0	1.0
Small town or rural	208950	167166 (59.6)	62 (41.6)	3.7 (2.8,4.6)	0.5 (0.3,0.7)	0.5 (0.4,0.7)
**Reason for testing**						
General group	90628	88116 (31.4)	32 (21.5)	3.6 (2.4,4.9)	1.0	1.0
HIV ISG	43517	39502 (14.1)	86 (57.7)	21.8 (17.2,26.4)	6.0 (4.0,9.0)	4.9 (3.2,7.5)
HIV TRG	8351	8144 (2.9)	10 (6.7)	12.3 (4.7,19.9)	3.4 (1.7,6.9)	2.6 (1.3,5.4)
STI risk group	201067	144694 (51.6)	21 (14.1)	1.5 (0.8,2.1)	0.4 (0.2,0.7)	1.1 (0.6,2.0)

HIV seroprevalence: the number of people living with HIV/AIDS per 10,000 individuals. All data are excluded 9,877 anonymous cases and 19,252 cases without full residential identification number (RID) or with incorrect RID. CI: Confidence Interval, OR: Odds Ratio. No. positive: the number of people living with HIV/AIDS.

HIV TRG: HIV test recommended group. HIV ISG: HIV infection suspected group. STI: sexually transmitted infection.

Table 3 presents the characteristics of HIV seroprevalence by reason for testing. Only in the HIV TRG, the male did not show significant difference from the female. Foreigners showed significantly higher seroprevalence in the General group (21.0 per 10,000 individuals) than in the STI risk group (14.1 per 10,000 individuals). There were no HIV positive foreigners identified in the HIV ISG and in the HIV TRG. Both groups displayed significantly higher seroprevalence than other groups in metropolitan cities (p < 0.05). For the HIV ISG, the seroprevalence was very high in the male (30.2 per 10,000 individuals), also the aged 30–39 years (37.9 per 10,000 individuals) and the aged 40–49 years (38.8 per 10,000 individuals) were very high.

**Table 3:** Comparison in HIV seroprevalences of visitors to public health centers by reason for HIV testing in Korea (2005).

	**General group**			**HIV ISG**			**HIV TRG**			**STI risk group**		
	Individuals (%)	HIV+	SP(95%CI)	Individuals (%)	HIV+	SP(95%CI)	Individuals (%)	HIV+	SP(95%CI)	Individuals (%)	HIV+	SP(95%CI)
**Total**	88116	32	3.6 (2.4–4.9)	39502	86	21.8 (17.2–26.4)	8144	10	12.3 (4.7–19.9)	144694	21	1.5 (0.8–2.1)
**Gender**												
Female	48628 (55.2)	8	1.6 (0.5–2.8)	13055 (33.0)	6	4.6 (0.9–8.3)	2359 (29.1)	3	12.7 (0.0–27.1)	128645 (88.9)	8	0.6 (0.2–1.1)
Male	39488 (44.8)	24	6.1 (3.6–8.5)	26447 (67.0)	80	30.2 (23.6–36.9)	5785 (70.9)	7	12.1 (3.1–21.1)	16049 (11.1)	13	8.1 (3.7–12.5)
**Age group**												
<20	6518 (7.4)	0	0.0	3230 (8.2)	3	9.3 (0.0–19.8)	951 (11.7)	0	0.0	4888 (3.4)	1	2.0 (0.0–6.1)
20–29	34290 (38.9)	12	3.5 (1.5–5.5)	14 673 (37.1)	15	10.2 (5.1–15.4)	1990 (24.4)	1	5.0 (0.0–14.9)	76440 (52.8)	7	0.9 (0.2–1.6)
30–39	22165 (25.2)	10	4.5 ((1.7–7.3)	10302 (26.1)	39	37.9 (26.0–49.7)	1492 (18.3)	1	6.7 (0.0–19.8)	36764 (25.4)	6	1.6 (0.3–2.9)
40–49	9275 (10.5)	9	9.7 (3.4–16.0)	5663 (14.3)	22	38.8 (22.6–55.1)	1380 (17.0)	6	43.5 (8.8–78.2)	21241 (14.7)	4	1.9 (0.0–3.7)
50–59	7352 (8.3)	0	0.0	2872 (7.3)	4	13.9 (0.3–27.6)	832 (10.2)	1	12.0 (0.0–35.6)	4140 (2.9)	3	7.2 (0.0–15.4)
60 ≤	8516 (9.7)	1	1.2 (0.0–3.5)	2762 (7.0)	3	10.9 (0.0–23.1)	1499 (18.4)	1	6.7 (0.0–19.7)	1221 (0.8)	0	0.0
**Nationality**												
Korean	80508 (91.4)	16	2.0 (1.0–3.0)	38710 (98.0)	86	22.2 (17.5–26.9)	8002 (98.3)	10	12.5 (4.8–20.2)	143277 (99.0)	19	1.3 (0.7–1.9)
Foreigner	7608 (8.6)	16	21.0(10.7–31.3)	792 (2.0)	0	0.0	142 (1.7)	0	0.0	1417 (1.0)	2	14.1 (0.0–33.7)
**Region**												
Metropolitan	42563 (48.3)	18	4.2 (2.3–6.2)	17691 (44.8)	53	30.0 (21.9–38.0)	3956 (48.6)	7	17.7 (4.6–30.8)	49080 (33.9)	9	1.8 (0.6–3.6)
Small town or rural	45553 (51.7)	14	3.1 (1.5–4.7)	21811 (55.2)	33	15.1 (10.0–20.3)	4188 (51.4)	3	7.2 (0.0–15.3)	95614 (66.1)	12	1.3 (0.5–2.0)

HIV TRG: HIV test recommend group. HIV ISG: HIV infection suspected group. STI: sexually transmitted infection

Individuals: the number of HIV tested individuals, HIV+: the number of people living with HIV/ADIS, SP (HIV seroprevalence): the number of people living with HIV/AIDS per 10,000 individuals.

CI: Confidence Interval

Among fourteen subgroups, the major parts taking tests were occupied by bar employees (31.2%) followed by the visitors requiring medical certificates (14.7%). Partners of HIV-infected individuals had the highest seroprevalence, followed sequentially by voluntary test takers (23.8 per 10,000 individuals), individuals referred by doctors (19.6 per 10,000 individuals), and prisoners (15.4 per 10,000 individuals). While there were no noticeable differences in the General group or the STI risk group after adjusted by gender, age, nationality, and region, individuals referred by doctors (adj. OR, 3.6; 95% CI, 1.7–7.5) and voluntary test takers (adj. OR, 5.7; 95% CI 2.8–11.8) had higher HIV seroprevalence than individuals for health checkup as a reference (Table 4).

**Table 4:** HIV seroprevalence of visitors to public health centers by reason for HIV testing in Korea (2005).

Group	Subgroup	No. of HIV tested individuals (%)	No. of positive	HIV Seroprevalence (95% CI)	Unadjusted OR (95% CI)	Adjusted OR (95% CI)
**Total**		280456	149	5.3 (4.5–6.5)	-	-

General group	Health Checkup	21598 (7.7)	9	4.2 (1.4–6.9)	1.0	1.0
	Medical Certificate	41358 (14.7)	19	4.6 (2.5–6.7)	1.1 (0.5–2.4)	1.0 (0.4–2.3)
	Prenatal Checkup	17159 (6.1)	2	1.2 (0.0–2.8)	0.3 (0.1–1.3)	1.2 (0.2–5.9)
	Others*	8001 (2.9)	2	2.5 (0.0–6.0)	0.6 (0.1–1.0)	0.8 (0.1–1.4)

HIV ISG	Referral by doctor	19359 (6.9)	38	19.6 (13.4–25.9)	4.7 (2.3–9.8)	3.6 (1.7–7.5)
	Voluntary test taker	20143 (7.2)	48	23.8 (17.1–30.6)	5.7 (2.8–11.7)	5.7 (2.8–11.8)

HIV TRG	TB patient	6822 (2.4)	3	4.4 (0.0–9.4)	1.1 (0.3–3.9)	0.9 (0.2–3.4)
	Prisoner	1295 (0.5)	2	15.4 (0.0–36.8)	3.7 (0.8–17.2)	2.5 (0.5–11.6)
	Partner of HIV-infected individual	27 (0.0)	5	1,852(387–3,317)	545 (169–999)	420 (110–999)

STI risk group	CSW	399 (0.1)	0	0.0	-	-
	Bar employee	87602 (31.2)	12	1.4 (0.6–2.2)	0.3 (0.1–0.8)	0.9 (0.4–2.2)
	Tea room employee	27353 (9.8)	2	0.7 (0.0–1.7)	0.2 (0.0–0.8)	0.8 (0.2–3.8)
	Massage parlor	1535 (0.6)	0	0.0	-	-
	Others**	27805 (9.9)	7	2.5 (0.7–4.4)	0.6 (0.2–1.6)	2.1(0.7–5.9)

CI: Confidence Interval, OR: Odds Ratio, TB: tuberculosis, CSW; commercial sexual worker.

HIV TRG: HIV test recommended group. HIV ISG: HIV infection suspected group. STI: sexually transmitted infection.

*Employees of motels, restaurants, and beauty salons, etc.

**One among four subgroup in STI risk group, but not specified.

## Discussion

HIV testing policy in Korea has been changed. Mandatory HIV testing was dominated until 1990's and it gradually has been moved to the voluntary HIV testing in hospitals or clinics. In 2005, hospitals or clinics conducted 55% of HIV screening tests in Korea, and detected 75% of the newly diagnosed HIV infections. [16] However, the HIV testing takers in PHCs occupied approximately 6% of the screening tests and comprised 19% (149 individuals or 5.3 per 10,000 individuals) of the newly diagnosed in Korea.

People aged 30–49 years, foreigners, metropolitan inhabitants, the HIV ISG, and the HIV TRG showed high seroprevalence. The high seroprevalence in the HIV ISG was due to suspicions by doctors and voluntary test takers experiencing symptoms or risky behaviors. The proportion of HIV-infected male who found in 2005 was 94% and all of them were infected by sexual contact. According to survey for HIV transmission route, the proportion of homosexual contact is 47.0%, heterosexual contact 52.8%, and others 0.2%. However, many males who were categorized into heterosexual contacts at the initial interview were later changed into homosexual contacts [17], and it is doubtful if homosexual contact might be a dominant route in Korea. Epidemiological studies for Japan and Thailand showed high prevalent in the aged 30–49 years for men having sex with men (MSM) [18,19]. The prevalent of the HIV ISG for the male and the aged 30–49 years might be due to MSM. However, we do not have any information about this, and it will be requested to study the sexual behavior in order to find the characteristics of HIV transmission route in Korea.

HIV seroprevalence in the aged 20–29 years was lower than those in older individuals. This is similar to the seroprevalence of herpes simplex virus type 2 (HSV-2) [20] but differs from HIV seroprevalence in the U.S., Russia, and the many European countries [21-23].

There are about 910,000 foreigners in Korea, and about 65% are from China, the Philippines, Thailand, Viet Nam, and Russia. [24]. Foreigners who are engaged in entertainment or sports for more than 3 months are required to take HIV tests in accordance with the Prevention Act for Acquired Immunological Deficiency Syndrome [7]. In this study, foreigners revealed significantly higher seroprevalence than native Korean (p < 0.0001). Two of 18 HIV-infected foreigners belonged to the STI risk group (14.1 per 10,000 individuals) and the others were included the General group (21.0 per 10,000 individuals). The major reason to take tests in the General group was to get medical certification, which contributed to the high seroprevalence. For the foreigners, medical certification approved by a compulsory medical checkup is required for a job or for residence, which may be a stricter requirement compared with the cases of Koreans. About 12% of the newly diagnosed HIV infections in PHCs were foreigners. In Japan, this is 13% in 2004, which the infectious status is similar to our result [25]. The Korean government provides foreigners with free of charge HIV testing, counseling, education, and medical tests in PHCs to prevent domestic population from new HIV infection, also to take care the foreigners' health.

The female in the STI risk group is to take a mandatory test every six months, but we found that the number of HIV test in them was less than twice per year. HIV-infected females from the STI risk group cannot be gainfully employed in businesses with routine testing. Therefore people employed in the high risk industries will likely take anonymous tests. If they are positive, they do not take due mandatory test, will leave the industry or go underground. The seroprevalence of female is lower in the STI risk group than in the General group, so that the change in testing policy for STI risk group need to be considered. A limitation of mandatory testing was demonstrated in Austria where HIV prevalence among registered CSWs is 0%, but it is 3.7% among illegal sex workers [26].

HIV infection is a major health problem in prisons around the world [27,28], and they show higher seroprevalence than other groups in Korea, although the reasons were not addressed in this study. HIV seroprevalence in metropolitan cities was higher than that in small towns or rural areas, and this result is similar to other countries [29].

The HIV seroprevalence in tuberculosis (TB) patients treated in PHCs was similar to that in the General group. Although Korea is under low HIV prevalence, and is under intermediate TB burden; the TB prevalence was estimated 12.3 per 10,000 in 2006 by WHO [30]. TB is the most common opportunistic infection in AIDS patients, and AIDS is a major cause of TB or pneumocystis-associated deaths in Korea [31,32]. However, HIV testing is not compulsory to TB patients in Korea, so it is difficult to find HIV prevalence among them. Fortunately, we could find HIV infections of TB patients in our study because TB patients in PHCs were recommended to take HIV tests.

By statute, the STI risk group and the HIV TRG are required to take mandatory testing at PHCs for the prevention of HIV transmission, and the HIV ISG to take voluntary tests for early detection. For the General group, HIV testing is free of charge, which leads low-income individuals to take test in PHCs. We can find the status of seroprevalence in each group and compare to characteristics of seroprevalence among groups, through taking mandatory testing and voluntary testing under the national HIV surveillance system. Our results were derived from the national HIV surveillance data collected by the HCIS. HIV seroprevalence of visitors to PHCs can be used for the evaluation of community health policy as well as national HIV/AIDS and STI policy. Therefore our results were highly significant to the study of HIV epidemiology in PHC testing takers.

Our study had several limitations. First, The General group included about 31% of the total testing takers in PHCs, and included major individuals with low income who were tested due to job, or welfare requirement. Therefore, patterns of HIV seroprevalence in the General group identified in this study may not be nationally representative. Secondly, the seroprevalence in the STI risk group may be underestimated because of the possibility that the individuals could undergo anonymous testing prior to mandatory testing. Third, the HIV seroprevalence (5.3 per 10,000 individuals) might be underestimated due to excluding the anonymous testing takers. In 2005, 35 HIV positive cases were detected among 9,877 anonymous tests at PHCs and their positivity were very higher for 35.4 per 10,000 tests. Fourth, getting medical certification for a job or residence application in the General group seems to be mandatory currently. This is more common in foreign workers.

## Conclusion

UNAIDS/WHO do not support mandatory testing of individuals except blood donors on public health grounds. Voluntary testing is more likely to result in behaviors change to avoid transmission HIV [33]. Also, our result revealed that voluntary testing might be superior in identification of HIV-infected individuals, since HIV seroprevalence was low in the STI risk group while high in the HIV ISG. In addition, the mandatory testing on STI risk group raises a conflicting issue between human rights of the group vs. prevention of STI transmission. It is currently discussed to change the policy on the national HIV testing in Korea. We suggest there are needs in Korea to develop a method encouraging more people to take voluntary tests at PHCs and also to expand anonymous testing centers and Voluntary Counseling and Testing Program (VCT) for general population to easily access to HIV testing.

## Competing interests

The authors declare that they have no competing interests.

## Authors' contributions

MKK, designed the study and write out drafts of the manuscript. JHL, performed the statistical analyses and participated in the study design and interpretation. CC, assisted with the study and data cleaning. EJL, assisted with the study and data collecting. SSK, conceived of the study and supervised all aspects of its implementation. All authors read and approved the final manuscript.

## Pre-publication history

The pre-publication history for this paper can be accessed here:


